# The influencing factors of health hazards of benzo[a]pyrene in cigarette mainstream smoke: The example of one brand in Beijing

**DOI:** 10.18332/tid/152419

**Published:** 2022-09-23

**Authors:** Junrui Chang, Qin Wang, Xiaoyan Dong, Tian Luo, Zhe Liu, Dongqun Xu

**Affiliations:** 1 National Institute of Environmental Health, Chinese Center for Disease Control and Prevention, Beijing, China

**Keywords:** benzo[a]pyrene, smoking regimens, tar concentration, smoking behavior, health hazard

## Abstract

**INTRODUCTION:**

The study focused on the influence of tar concentrations, smoking regimen, and smoking behavior, on benzo[a]pyrene (B[a]P) emission from cigarette mainstream smoke and related health hazards to determine the key factors influencing B[a]P reduction and protection of the smoker’s health.

**METHODS:**

A locally popular brand of cigarettes in Beijing was selected with tar concentrations of 1, 3, 5, 8, 10, and 11 mg/cigarette. Two different machine smoking regimens, the Canada Intense (HCI) regimen and the International Organization for Standardization (ISO) regimen, were adopted to collect the cigarette mainstream smoke. The B[a]P emission concentrations were then measured by gas chromatography and mass spectrum.

**RESULTS:**

The average B[a]P emission was 8.14–17.6 ng/cigarette for the HCI regimen and 0.92–3.46 ng/cigarette for the ISO regimen. As expected, the tar concentrations and B[a]P emissions exhibited a positive relation in both the HCI and ISO regimens, the cancer risk and non-cancer risk increased with an increase in tar concentrations for both the ISO and HCI regimens, and the smoking behavior also affected the B[a]P emissions with a tendency of VB (ventilation blocking) > HVB (half ventilation blocking) > DP (deep puff), under the same smoking regimen. Under the same conditions, the cancer risk and non-cancer risk in men were 1.19 and 1.11 times, respectively, higher than in women.

**CONCLUSIONS:**

The smoking regimen influences the B[a]P emission relatively more than the cigarette tar concentration and smoking behavior. The cancer risk and non-cancer risk are higher in men than in women that possibly due to longer smoking duration and greater smoking intensity.

## INTRODUCTION

Mainstream cigarette smoke contains more than 7000 chemical components, all of which are harmful toxicants. Globally, there were 1.14 billion current smokers and 7.41 cigarettes were consumed in 2019, and the average smoking rates were approximately 27.5% and 37.3% in men and women, respectively^1^. From 1990 to 2019, 7.69 million deaths and 200 million disability-adjusted life-years came from tobacco use and related economic costs exceed 1 trillion US dollars^2,3^. Carcinogenic constituents are generated in combustion during cigarette smoking, these carcinogenic constituents are inhaled through the respiratory system. Smoking can cause cancers of multiple organs, such as the hematopoietic system, cervix, and colorectal organ but lung cancer is the main one of them^4^. In China, there was more than a third of the world’s tobacco consumption which accounted for nearly one-third of lung cancer cases in the world1, with the burden of lung cancer being the highest^5^. In Beijing, the smoking rate in adults was approximately 22.3%, and there were about 4 million smokers in 2016^6^. More than 70 agents are classified as carcinogens for animals and humans by the International Agency for Research on Cancer (IARC)^7^. Polycyclic aromatic hydrocarbon (PAH) is a type of carcinogen that can be produced from cigarette smoke, as one of PAHs, Benzo[a]pyrene (B[a]P) was classified as a carcinogenic agent for humans by IARC and is a representative pollutant of PAHs, and is regarded as a representative marker for other PAH constituents in cigarettes^8^ and present in large quantities in cigarette smoke; it contributes to approximately 50% of total cancer risk of PAHs^9^.

Several factors can affect the emission of pollutants from cigarette smoking. For instance, the cigarettes of different brands release different quantities and types of pollutants due to different raw materials used and manufacturing processes^9^. In addition, there were gender differences in tobacco consumption, in general, the prevalence of current use of smoking tobacco among males was higher than in females and 80% of the deaths from smoking were among males; and smoking was the first cause of death in males, amongst the 87 risk factors included in GBD 2019^2^. Smokers adopt different puffing depths when smoking; the International Organization for Standardization (ISO) and Health Canada Intense (HCI) smoking regimens represent two different kinds of human puffing style^10^, referred to as puffing topographies. The HCI regimen represents an intense smoking scenario that produces more pollutants, and the ISO regimen represents a regular smoking scenario that produces relatively less pollutants^11^. According to a WHO report, no machine smoking regimen can represent all human smoking characteristics, and both the ISO and HCI regimens should be considered in pollutant emission and hazard assessment^12^. Tar concentrations of cigarettes can affect pollutant emission and related health hazards^13^. In addition, other smoking characteristics also affect the cancer risk of smokers. For example, some studies have indicated that the smoking duration and intensity are associated with lung cancer, with the smoking duration showing a stronger effect than smoking intensity. However, other studies have reported that smoking intensity has a more profound effect on hazardous substance emissions and cancer risk^14^. Other smoking behaviors such as ventilation blocking (VB), half ventilation blocking (HVB), and deep puffing (DP) also affect the emission of pollutants and carcinogenic agents from cigarette smoke. The design of the ventilation system in cigarette filters can effectively reduce the absorption of toxicants, but some people block the ventilation partly or wholly with their finger when smoking^15^. Currently, the health effects of this kind of behaviors on smokers are unknown.

Some studies have studied the influence of smoking characters (tar concentration of cigarettes, the puffing topographies, smoking behavior and smoking habit) on pollutant emission and related health hazards^16,17^, but the study of Hirayama^18^ found that there were different cancer risks of smoking in different countries, even when people had the same cigarette exposure level. In this article, local cigarettes of a brand in Beijing were selected which contained different tar concentrations so as to analyze the influence of local cigarettes and smoking characteristics on the B[a]P emission levels and cancer risk, and non-cancer risk of local people, which helps to ascertain the health hazards of B[a]P inhalation from cigarette smoke and the importance of decreasing the B[a]P release from a cigarette.

## METHODS

### Selection and processing of cigarettes

A local popular brand of cigarettes in Beijing was selected for this study, with different tar concentrations of 1, 3, 5, 8, 10, and 11 mg/cigarette. There were 2 rows of parallel ventilation holes in the middle of the cigarette filter. The cigarettes were assigned to three smoking behaviors according to different blocking status, namely deep puff (ventilation holes were not blocked), half ventilation blocking (one row of holes was completely blocked with tape), and ventilation blocking (two rows of holes completely blocked with tape).

### Sampling of the mainstream smoke of cigarettes

All the cigarettes and glass-fiber filter pads (Cambridge Filter Pad [CFP], Whatman, UK) were kept for 48 h under 22^o^C and 60% RH according to the ISO 3308:2000 (routine analytical cigarette-smoking machine-definitions and standard conditions) and ISO 3402:1999 (atmosphere for conditioning and testing tobacco and tobacco products). A linear smoking machine with 20-channels (CERULEAN SM450, UK) was used and cigarettes were smoked as two regimens: 1) the ISO regimen, which included 35 mL puff volume, 60 s puff interval, and no blocking of the filter ventilation holes; and 2) the HCI regimen, which included 55 mL puff volume, 30 s puff interval, and 100% blocking of the filter ventilation holes. The total particulate matter of the mainstream smoke was collected on filter pads. Each pad collected for five cigarettes under different smoking regimens and behavior.

### Determination of B[a]P emission levels

B[a]P absorbed onto a filter pad was extracted through ultrasonic extraction. The filters were placed in a centrifuge tube, to which 16 mL of solvent consisting of dichloromethane: benzene: acetonitrile (2:1:1, Fisher, America) was added, and then, ultrasonic extraction was conducted for 20 min, repeating this step three times; the extracts were combined and blown to a nearly dry state using nitrogen, then dissolved with 1 mL solvent consisting of dichloromethane, benzene, acetonitrile and filtered with syringe filters. The B[a]P concentrations were then detected by gas chromatography and mass spectrometry (GC-MS; Trace 1300 ISQ QD, Thermo Fisher).

### Assessment of the health hazard of B[a]P emission from local cigarettes

*Calculation of the exposure concentration of B[a]P in cigarettes*

The exposure concentrations represent the exposure level of the pollutants related to the health hazard. According to the USA EPA^19^, the exposure level of a smoker was affected not only by the pollutants contained in the cigarette but also the number of cigarettes consumed in a day and the smoking duration. In addition, the inhalation rate, which is one of the smoker’s characteristics, influences the exposure concentration. The exposure concentration of B[a]P was calculated as follows:


EC=(C×CpD×ED×EF)/(IR×AT) (1)

where *EC* is the exposure concentration (ng/m^3^); C is the B[a]P concentration per cigarette (ng/cigarette); *CpD* is the number of cigarettes per day; *ED* is exposure duration (years); *EF* is exposure frequency (days/year); and *IR* is inhalation rate (m^3^/day). According to the Exposure Factors Handbook of Chinese Population published in 2016^20^, *IRs* of 16.9, 18.8, and 15.1 m^3^/day for the general people, men, and women, respectively, were adopted. *AT* is the lifetime, 70×365 days. A study reported that the average age when the individuals started smoking was 19.1 years in Beijing, and this age for male and female smokers was 18.9 and 24.7 years, respectively. The *ED* was 50.9, 51.1, and 45.3 years for the general people, men, and women, respectively, when the lifetime duration was 70 years, and the *CpDs* were 15.2, 15.4, and 11.7 for general people, men, and women, respectively^21^.

*Assessment of the cancer risk of B[a]P emission in cigarettes*

Excess lifetime cancer risk (*ELCR*) is commonly used to characterize cancer risk. According to USA EPA guidelines^22^, the cancer risk is not accepted when it is >10^-6^. The equation used is as follows:


ELCR=EC×IUR (2)

where *IUR* is inhalation risk (ng/m^3^). Referring to the cancer risk, B[a]P exposure was 1 ng/m^3^ in 70 years lifespan; for the same pollutant, several institutes reported different values due to the different sources of data. In our study, the value of the California EPA of 1.1×10^-6^ was adopted^22^.

*Assessment of the non-cancer risk of B[a]P exposure from Beijing local cigarettes*

The non-cancer risk can be represented using the hazard quotient (*HQ*), which refers to the value of a single pollutant. The acceptable HQ level should be <1. The formula used is as follows:


HQ=EC/Rfc (3)

Reference concentration (*Rfc*) is the toxicology value acquired from animal experiments^23^. The Rfc of B[a]P was 2×10^-6^ mg/m^3^.

### Statistical analysis

MS Excel and SPSS 19.0 were used for statistical analysis and drawing, and also to calculate the exposure concentration, the risk of cancer and non-cancer. The K-S analysis was employed to determine the type of distribution data. The average and standard deviation were calculated to represent the central tendency and dispersion tendency. Pearson correlation was used to analyze the correlation of tar concentration and B[a]P emission level, and a t-test was used to determine the significance of the correlation.

## RESULTS

### The difference in the B[a]P emissions between ISO and HCI smoking regimens

The results revealed that, with different tar concentrations in cigarettes of the same brand, the B[a]P emission levels varied (Table 1). The average B[a]P emission was 8.14–17.6 ng/cigarette in the HCI regimen and 0.92–3.46 ng/cigarette in the ISO regimen. The B[a]P emission of the HCI regimen was 5.09–9.29 times higher than that in the ISO regimen; the cigarettes of 3 mg/cigarette and 11 mg/cigarette showed the highest and lowest multiples, respectively.

**Table 1:** B[a]P emissions of cigarettes containing different tar concentrations under the HCI and ISO regimens (N=60)

*Tar (mg)*	*HCI regime*		*B[a]P/tar*	*ISO regime*		*B[a]P/tar*	*Multiple (HCI/ISO)*
	*n*	*Concentration I/ISO) (ng/cigarette)*		*n*	*Concentration (ng/cigarette)*		
1	5	8.14±0.63	8.14	5	0.92±0.13	0.92	8.85
3	5	10.5±0.61	3.50	5	1.13±0.16	0.38	9.29
5	5	11.8±1.15	2.36	5	1.33±0.19	0.27	8.87
8	5	14.0±0.99	1.75	5	1.67±0.25	0.21	8.38
10	5	16.3±0.48	1.63	5	2.78±0.38	0.28	5.86
11	5	17.6±0.67	1.60	5	3.46±0.96	0.31	5.09

### Relationship between the tar concentration and B[a]P emission, and the health hazard related to different tar concentrations

**Figure 1:**
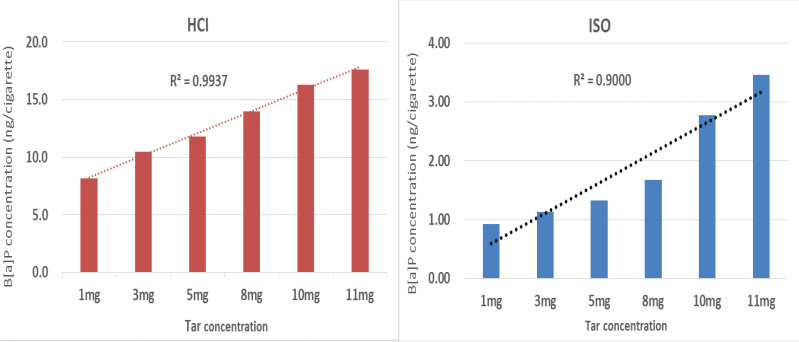
Relationship between the tar concentration and B[a]P emission

The tar concentration and B[a]P emission level had a positive relationship in both the HCI and ISO regimens (Figure 1). The coefficient of determination in the HCI and ISO regimens was 0.9937 and 0.9000, respectively, and the corresponding correlation coefficients were 0.9968 and 0.9487, both regimens showed statistical significance (p<0.05). Compared with the 1 mg/cigarette tar concentration, the B[a]P emission level increased by 29%, 50%, 72%, 100.2%, and 116.2% for cigarettes with tar concentrations of 3, 5, 8, 10, and 11 mg/cig, respectively, in the HCI regimen. Similarly, the B[a]P emission level increased by 22.9%, 44.6%, 81.5%, 202.2%, and 276% in the ISO regimen.

**Figure 2:**
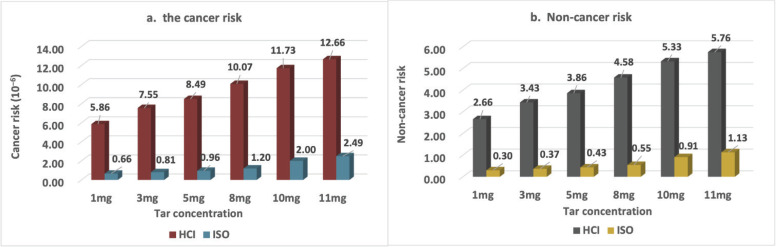
The risk of cancer and non-cancer related to the cigarettes containing different tar concentrations

For DP, the cancer risk was always >10^-6^ irrespective of the tar concentration of the HCI regimen (Figure 2a) and ISO regimen, the cancer risk increased with an increase in the tar concentration. When the tar concentration ≤5 mg/cigarette, the cancer risk was <10^-6^, but when the tar concentration was ≥8 mg/cigarette, the cancer risk was >10^-6^. The HQ was always >1 for the HCI regimen (Figure 2b). For the ISO regimen, the non-cancer risk was observed at 11 mg/cigarette (highest tar concentration of cigarettes) but not at low tar concentrations.

### Effects of different smoking behavior on B[a]P emission and the related health hazard

**Figure 3:**
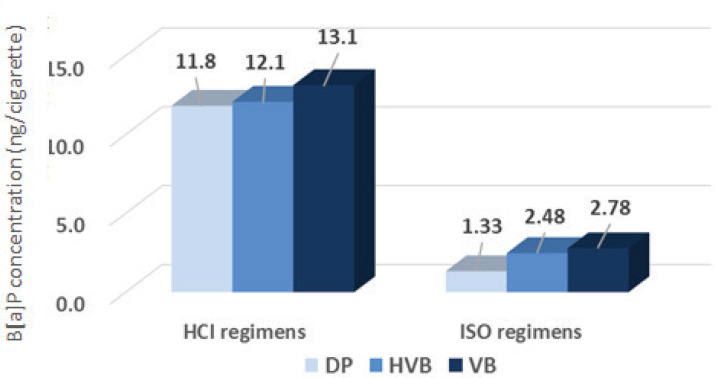
Comparison of the levels of B[a]P emission under different smoking behaviors

Figure 3 shows that the B[a]P emission ranged from 1.33 ng/cigarette to 2.78 ng/cigarette in the ISO regimen with DP, HVB and VB, and 11.8 ng/cigarette to 13.1 ng/cigarette in the HCI regimen, for the B[a]P emission level VB>HVB>DP for the same smoking regimen. Moreover, under the same smoking behavior, the HCI regimen was 8.87, 4.88 and 4.71 times higher than that of the ISO regimen.

**Figure 4:**
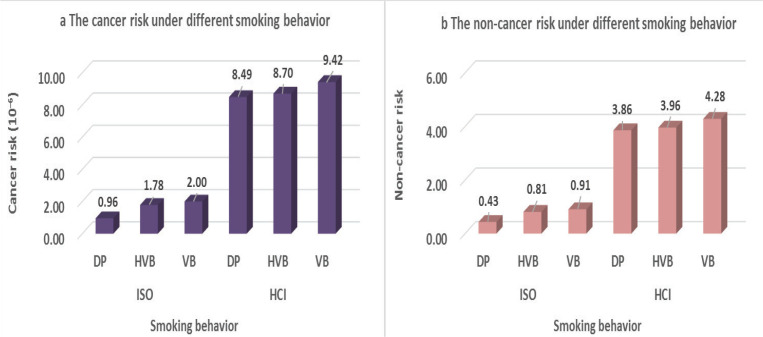
The risk of cancer and non-cancer related to different smoking behaviors

The cancer risk assessment related to different smoking behaviors is shown in Figure 4. Taking the 5 mg tar concentration cigarette as an example, the two smoking regimens showed the same tendency, with the DP and VB related to the lowest and highest cancer risk, respectively. Under the ISO regimen, the cancer risk of HVB and VB was 1.85 and 2.08 times higher than that of DP. For the HCI regimen, the cancer risk of HVB and VB was 1.02 and 1.11 times higher than that of DP. While analysis of the non-cancer risk, the same results were obtained. For both the ISO and HCI regimens, the non-cancer risk indicated VB>HVB>DP. For the ISO regimen, the non-cancer risk of HVB and VB was 1.97 and 2.11 times higher than that of DP. For the HCI regimen, the cancer risk of HVB and VB was 1.02 and 1.11 times higher than that of DP.

### Effect of different smoking habits on health hazards in males and females

**Figure 5:**
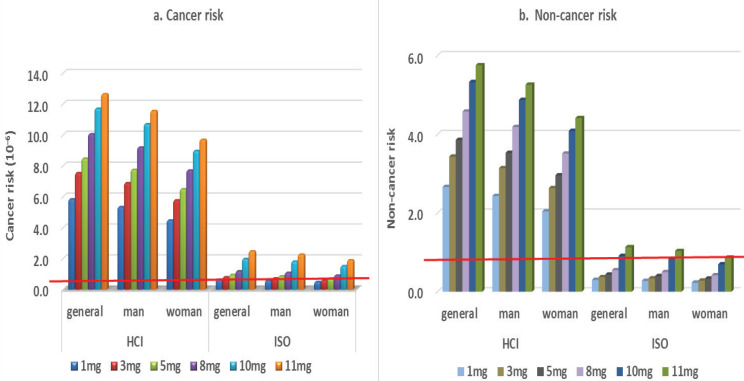
The cancer risk and non-cancer risk under the HCI and ISO regimens in different gender

The general people, men, and women have different smoking habit, their smoking habit parameters were used to calculate the cancer risk and non-cancer risk under two smoking conditions (Figure 5a). When the DP condition was adopted, the cancer risk was >10^-6^ for all people on the HCI regimen. On the ISO regimen, there was a potential cancer risk for general people and men when the tar concentration of cigarette was >8 mg/cigarette, and for women there was potential cancer risk when the tar concentration was >10 mg/cigarette.

The non-cancer risks were always >1 in the HCI regimen, irrespective of the tar concentrations (Figure 5b). For the ISO regimen, the non-cancer risks for general people and men were >1 when they smoked the cigarettes containing tar of 11 mg/cigarette. Women’s HQ was always <1 even when smoking cigarettes with high tar content. The non-cancer risk of men was 1.11 times higher than that of women.

## DISCUSSION

Studies have shown that the B[a]P emission levels for cigarettes containing 6 and 1 mg/cigarette tar concentrations are 5.05–5.37 and 0.97–1.01 ng, respectively, under the ISO regimen^24^. The B[a]P emission levels are 11.1–16.8 ng/cigarette under the HCI regimen and the B[a]P emission level for the HCI regimen was 2.3 and 2.5 times higher than that for the ISO regimen^25^ whose results are in accordance with our results. Moreover, in our study, except for the cigarette containing 3 mg/cigarette tar concentration, there was a trend of a higher HCI/ISO ratio of B[a] P emission at lower tar concentrations, and all the differences were statistically significant (p<0.05), the results were similar to those of the Bian et al.^26^ study. The above results indicate that more B[a]P emissions were recorded under the HCI regimen than under the ISO regimen.

According to USA Environmental Protection Agency, the percentile should be employed while assessing the health risk^19^, for example, the average, percentile 10th, and percentile 90th should be adopted to indicate the health risk of average, upper limit and lower limit, respectively^27^, which cover the possible range of health hazards. Besides, some studies have indicated that the data from the HCI regimen should be used as an upper limit and that the data from the ISO regimen should be employed as a regular value^24,28^. In addition, the smoking behavior of the same person could be changed by varying the nicotine concentration of cigarettes^29^. While smoking cigarettes with lower or higher nicotine concentrations, smokers tend to take a deep or shallow puff. The ISO and HCI regimens represent the state of shallow and DP, respectively. In China, cigarettes with tar concentration of ≤10 and >10 mg/cigarette are considered lower and higher tar cigarettes, respectively^30^. The cigarettes of different brands release different types and quantities of pollutants due to the use of different raw materials, manufacturing processes, and filter materials. In our study, a single brand in Beijing with 1–11 mg/cigarette tar concentration was analyzed, and the results indicate that the B[a]P emissions increases with an increase in tar concentration. Other studies have also found that the toxicants emissions generally change with the rank order of cigarette tar. For instance, the B[a] P emission increased by 41.2% in 6 mg tar cigarettes compared with 1 mg tar cigarettes^31^.

Our results indicate that the studied cigarette brand had a cancer risk and non-cancer risk irrespective of the tar concentration under the HCI regimen. Our study also revealed that the cancer risk and non-cancer risk increased with an increase in the tar concentration in cigarettes for both the ISO and HCI regimens, which indicates that cigarettes containing low tar concentration could potentially reduce the health hazards to smokers compared with cigarettes containing high tar concentration, the results agree with the findings of other studies^32^ but many studies reported that the health hazard of cigarettes has no relationship with tar content^33^. Some studies reported the cancer risk and non-cancer risk to the population by using HCI data as the upper limit and by taking the ISO data as the mean, but did not mention the tar concentrations^34^.

Smokers can adopt other approaches to ensure control over their nicotine intake. For instance, blocking the hole with their mouth or fingers when smoking leads to the inhalation of excessive nicotine for satisfying their smoking needs^35^. Simultaneously, the intake of pollutants such as B[a]P increases with an increase in the nicotine concentration. The study of Bates et al.^36^ indicated that compensatory smoking can lead to deep puffing which minimizes the benefits of reduced carcinogenic agent levels. Comparing the B[a]P emission under different smoking behaviors and taking the cigarette of 5 mg/cigarette as an example, our results show that the smoking behavior can affect the B[a]P emission, but the smoking regimen influences the B[a]P emission relatively greater.

Factors related to smoking habits include smoking duration and intensity, which refer to the smoker’s activity characteristics. Some studies have indicated that exposure to cigarette pollutants is different for every smoker due to their different smoking habits^37^. Even for the same smoker, the exposure to pollutants can vary on different days, leading to different health hazards. Many studies have shown differences in the smoking puff depth and smoking habit between men and women^21^. Our study shows that women’s cancer risk is lower than that of men due to the smaller smoking duration and lower cigarette consumption. Under the same smoking regimen, the smoking behavior and tar concentration (5 mg/cigarette), the cancer risk of men was 1.19 times higher than that of women. Other studies have found similar results, stating that the overall cancer rate in men is 2.5 times greater than that in women^38^. These results were obtained because men smoked for a longer duration and consumed more cigarettes than women. When the smoking habit was similar between men and women, their cancer rates were closer. The non-cancer risk also showed the same tendency.

These results showed that irrespective of tar concentrations, cigarettes posed a cancer risk and non-cancer risk for all people under the HCI smoking condition. Under the ISO smoking condition, the cancer risk and non-cancer risk were related to the smoking habit and increased with an increase in the cigarette tar concentration. For the same smoking regimen, the same smoking behavior, and the same tar concentration (5 mg/cigarette), the cancer risk and non-cancer risk in men was higher than that in women due to the longer smoking duration and the larger number of cigarettes consumed.

### Limitations

Any smoking regimen cannot represent the real state of people smoking (WHO, 2016); the puff topographies and smoking behavior differs from person to person, even for the same person these factors can vary. This study was limited by the fact that we considered different smoking regimens to cover the actual intake and health hazards of the smokers.

Cigarette combustion can produce thousands of toxicants, many of which are hazardous for human health, for example B[a]P. In this study, we only estimated the B[a]P emission and related health hazards, which do not cover the entire spectrum of health hazards. In addition, the effect of these compounds (with respect to their interaction with humans or effect on the health) in entirety differs from their independent effects. Therefore, it is necessary to assess the synthesized health hazard of different pollutants in the future, but this is difficult during health hazard assessment. In our study, we assumed that B[a]P can be completely absorbed, in fact, humans only absorb part of the B[a]P from a cigarette. This assumption possibly overestimates the health hazards.

## CONCLUSIONS

The tar concentration and B[a]P emission level showed a positive relation in both the HCI and ISO regimens; the B[a]P emission level was higher in the HCI regimen than in the ISO regimen. The smoking regimens, cigarette tar concentration, smoking behavior all can influence the B[a]P emission level, consistent with the study of Vu et al.^8^. The cancer risk and non-cancer risk increases with an increase in the cigarette tar concentration for both the ISO and HCI regimens.

The possibility of occurrence of the cancer risk and non-cancer risk increased with an increase in the tar concentration. The B[a]P emission showed a tendency of VB > HVB > DP under the same smoking regimen and the cancer risk and non-cancer risk demonstrated the same tendency: VB > HVB > DP. There was cancer risk and non-cancer risk regardless of the tar content under the HCI smoking regimen. For the ISO regimen, the cancer risk and non-cancer risk of cigarettes was related to the tar concentration, the health risk was observed when the cigarette tar was ≥8 mg/cigarette.

The cigarette tar, smoking regimen, smoking behavior influence the B[a]P emission levels, but the effect of smoking regimen on the B[a]P emission level was the highest.

The cancer risk and non-cancer risk of smoking were influenced by the gender of the smoker. Under the same smoking regimen, smoking behavior, and cigarette tar, the cancer risk and non-cancer risk were higher in men than in women, possibly due to the longer smoking duration and larger number of cigarettes consumed by men than by women.

## Data Availability

The data supporting this research are available from the authors on reasonable request.
